# Maternal Prepregnancy BMI and Glucose Level at 24–28 Gestational Weeks on Offspring's Overweight Status within 3 Years of Age

**DOI:** 10.1155/2017/7607210

**Published:** 2017-01-30

**Authors:** Weiqin Li, Leishen Wang, Nan Li, Wei Li, Huikun Liu, Shuang Zhang, Gang Hu, Junhong Leng

**Affiliations:** ^1^Tianjin Women's and Children's Health Center, Tianjin 300070, China; ^2^Department of Epidemiology, Capital Institute of Pediatrics, Beijing 100020, China; ^3^Chronic Disease Epidemiology Laboratory, Pennington Biomedical Research Center, 6400 Perkins Road, Baton Rouge, LA 70808, USA

## Abstract

*Objective*. To examine the relative impact of maternal prepregnancy body mass index (BMI) and glucose level at 24–28 gestational weeks on offspring's overweight status from birth to 3 years of age in China.* Methods*. Health care records of 21,354 mother-child pairs were collected. The single and joint associations of maternal prepregnancy BMI and glucose level at 24–28 gestational weeks with 0–3-year-old offspring's overweight status were assessed.* Results*. The odds ratios (95% confidence intervals) of offspring's macrosomia at birth and overweight/obesity at the 12th month, 24th month, and 36th month were 1.12 (1.11–1.13), 1.05 (1.04–1.06), 1.07 (1.06–1.08), and 1.11 (1.10–1.12) for each 1-unit increase (km/m^2^) in maternal prepregnancy BMI and 1.13 (1.10–1.17), 1.01 (0.99–1.03), 0.99 (0.96–1.01), and 1.00 (0.97–1.02) for each 1-unit increase (mmol/L) in maternal glucose level at 24–28 gestational weeks, respectively. The positive association of maternal glucose level with macrosomia at birth was similar between prepregnancy normal weight (BMI < 24 kg/m^2^) and overweight (BMI ≥ 24 kg/m^2^); however, the positive association of high maternal glucose level with childhood overweight was only seen among prepregnancy normal weight mothers but not among overweight mothers.* Conclusions*. The impact of maternal gestational hyperglycemia on offspring's overweight before 3 years of age can be modified by prepregnancy BMI.

## 1. Introduction

Childhood obesity is a global problem with the prevalence of 6.7% in 2010 worldwide [1]. Recent studies have shown that excessive weight gain and/or overweight in the first several years of life would increase the risks of subsequent obesity and unfavorable metabolic outcomes in childhood, adolescence, and adulthood [2–4]. Identifying maternal risk factors in the prepregnancy or prenatal period may help develop early intervention strategies for primordial obesity prevention [2].

The prevalence of maternal prepregnancy overweight/obesity has increased in recent years, and about one-fourth of women in China had overweight or obesity at prepregnancy in 2009-2010 [5]. Maternal prepregnancy overweight/obesity increases the risks of poor fetal outcomes including macrosomia at birth and overweight at childhood [6, 7] and the risks of maternal outcomes including gestational diabetes mellitus (GDM) [8]. GDM is another independent risk factor for offspring's macrosomia at birth. Some studies have reported that GDM has a stronger impact than maternal prepregnancy obesity on offspring's macrosomia at birth [9, 10]. However, some other studies suggested that a higher risk of macrosomia was associated with maternal prepregnant obesity [6, 7]. Until now, the relative impact of maternal obesity and hyperglycemia on the risk of macrosomia is unclear. In addition, it is important to assess the impacts of maternal prepregnant obesity and hyperglycemia during pregnancy on the risk of offspring's overweight at the first several years of life [11, 12]. Therefore, the aim of the present study was to examine the relative effect of maternal prepregnancy body mass index (BMI) and glucose level at 24–28 weeks of gestation on offspring's overweight status from birth to 3 years of age in Tianjin, China.

## 2. Methods

### 2.1. Study Sample

Tianjin is the fourth largest city with over 12.3 million residents in northern China, and 4.43 million residents lived in six central urban districts and one new urban district (Tanggu) in 2009. The prenatal care and children's health care in Tianjin are routine, with a three-tier care system consisting of more than 300 primary hospitals, 6 district-level Women and Children's Health Centers (also including secondary hospitals), and a city-level Women and Children's Health Center (also including tertiary hospitals). In Tianjin, all pregnant women are registered within the first 12 gestational weeks at primary hospitals where they receive antenatal care until the 32nd gestational week unless they have a high-risk pregnancy and then are referred to a secondary or a tertiary hospital for antenatal care till delivery. All children are given health examinations at birth, during postnatal period (<42 days after birth), and at infancy, early childhood, and preschools. Health care records for both pregnant women and their children have been collected and available in electronic form since 2009 [13]. The Pregnant Women Health Records include general information (date of first antenatal care visit, age, ethnicity, education, abortion history, last menstrual period, smoking habits, etc.), clinical measurements (height, weight, and blood pressure for each antenatal care visit, gynecological examinations, ultrasonography, glucose challenge test [GCT] at the 24–28 weeks of gestation, and other lab tests), pregnancy outcomes (date of delivery, delivery modes, labor complications, etc.), and postnatal period examinations (<42 days after delivery). The Children Health Records include information at birth (date of birth, sex, gestational week of birth, birth weight, birth length, etc.) and during postnatal period (family history of diseases, weight, and recumbent length) and health examinations at infancy (each three months during the first 12 months), early childhood (each six months during 1–3 years), and preschool period (each year during 3–6 years).

Between January 2009 and December 2010, a total of 43,446 women with single-pregnancy in six central urban districts and one new urban district (Tanggu) were registered for pregnancy and attended their first antenatal care at primary care hospitals within the first 12 weeks of gestation. Finally, the present study included 21,354 mother-child pairs (49.2%) with all the information of maternal height, prepregnancy weight, glucose level, and child weight and length/height at birth, the 12th month, 24th month, and 36th month. Compared with the mothers excluded, mothers included in the present analysis had younger age (27.9 versus 28.2 years old, *p* < 0.001), similar prepregnancy BMI (22.2 versus 22.2 kg/m^2^, *p* = 0.46) and glucose level at 24–28 weeks of gestation (6.59 versus 6.60 mmol/L, *p* = 0.82), and similar rate of male offspring (51.5% versus 52.4%, *p* = 0.065). The Ethics Committee for Clinical Research of Tianjin Women and Children's Health Center approved the study and analysis plan. Tianjin Women and Children's Health Center agreed to waive the need for written informed consent from all participants involved in our study because we used the electronic dataset from health care records.

### 2.2. Measurements

Mothers' weight and height at the first antenatal visit were measured in light clothing and without shoes by using a beam balance scale (RGZ-120, Jiangsu Suhong Medical Instruments Co., China). Body mass index (BMI) was calculated by dividing weight in kilograms by the square of height in meters and was categorized as underweight (BMI < 18.5 kg/m^2^), normal weight (18.5 kg/m^2^ ≤ BMI < 24 kg/m^2^), overweight (24 kg/m^2^ ≤ BMI < 28 kg/m^2^), or obese (BMI ≥ 28 kg/m^2^) according to the standard of Working Group on Obesity in China [14]. Because the average (range) gestational weeks at the first antenatal visit were 10.3 (2.6–12.9) gestational weeks and a previous study reported that there was a high correlation between self-reported prepregnancy weight and the weight recorded at the first visit [15], BMI at the first antenatal visit was treated as prepregnancy BMI.

All pregnant women underwent a gestational diabetes mellitus (GDM) screening test at 24–28 weeks with a 1-hour 50 g oral glucose challenge test (GCT). Plasma glucose levels were measured using an automatic analyzer (TBA-120FR; Toshiba, Japan) 60 minutes after the ingestion of 200 ml of 25% glucose solution. Glucose level ≥ 7.8 mmol/L was defined as the high-risk marker for GDM and a dichotomous variable was created to reflect this situation—yes versus no.

Children's weight and length/height were measured at birth, the 12th month (±2 months), 24th month (±2 months), and 36th month (±2 months). Weight was measured to the nearest 0.01 kg by using a digital scale (TCS-60, Tianjin Weighing Apparatus Co., China). Length was measured to the nearest 0.1 cm by using a recumbent length stadiometer (YSC-2, Beijing Guowangxingda, China). The electronic data of maternal and offspring's weight and length/height were validated [5]. *Z* scores (standard deviation [SD] scores) were calculated independent of sex and age—that is, (measurement minus population mean)/population SD—in each infant for BMI at months 12, 24, and 36 based on the standards of WHO growth reference [16]. Overweight was defined as BMI above the 85th percentile (≥1.035 *Z* score), and obesity was defined as BMI above the 95th percentile (≥1.645 *Z* score) [16]. Macrosomia was defined as birth weight ≥ 4000 grams. The information of feeding mode during the first 6 months was collected and divided into 4 categories because some mothers have to go back to work after 4 months' maternity leave in China: exclusive breast-feeding (exclusively breast-feeding more than 6 months), mixed breast and formula feeding, weaned from breast-feeding (exclusively breast-feeding but less than 6 months), and exclusive formula feeding. There was significant association between childhood obesity/overweight and mode of feeding. Children who fed on exclusive breast milk were less likely to be overweight, while children fed on exclusive formula milk were more likely to be overweight (Supplementary Table  1, in Supplementary Material available online at https://doi.org/10.1155/2017/7607210).

### 2.3. Statistical Analyses

The general characteristics of both mothers and children based on different categories of maternal prepregnancy BMI and glucose level during pregnancy were compared by using the General Linear Model and chi-square test. Logistic regression models were used to estimate the single and joint associations of prepregnancy BMI and glucose level during pregnancy as both categorical (prepregnancy BMI: <18.5, 18.5 ≤ BMI < 24 [reference], 24 ≤ BMI < 28, and ≥28 kg/m^2^; glucose level: <7.8 [reference] and ≥7.8 mmol/L) and continuous variables with the risks of macrosomia at birth and childhood overweight/obesity during the early years of life. We included two models in macrosomia analyses at birth: Model 1 adjusted for maternal age, ethnicity, education, smoking status, history of abortion, systolic blood pressure at 24–28 gestational weeks, gestational age at delivery, and child sex; Model 2 adjusted for variables in Model 1 and further adjusted for glucose level (in prepregnancy BMI analyses) or prepregnancy BMI (in glucose level analyses). In childhood overweight/obesity analyses, there were also two models: Model 3 adjusted for variables in Model 1 and further adjusted for birthweight and mode of infant feeding; Model 4 adjusted for variables in Model 2 and further adjusted for birthweight and mode of infant feeding. The criterion of statistical significance was <0.05 (for two-sided tests). All statistical analyses used SAS for Windows, version 9.3 (SAS Institute, Cary, NC).

## 3. Results

The general characteristics of both mothers and children based on maternal prepregnancy BMI and glucose levels at 24–28 gestational weeks are presented in Table 1. Overall, the prevalence of prepregnancy underweight, normal weight, overweight, and obesity was 11.2%, 63.7%, 19.0%, and 6.1%, respectively. There were 4,033 (18.9%) women with glucose level ≥ 7.8 mmol/L at 24–28 gestational weeks. Women with prepregnancy overweight or obesity and glucose level ≥ 7.8 mmol/L at 24–28 gestational weeks were older, had higher systolic blood pressure (SBP), were more likely to have a history of abortion, and had smaller gestational age at delivery (All *P* values < 0.05).

Children born to mothers with higher prepregnancy BMI had greater mean values of body weight and length/height from birth to 36 months and higher prevalence of macrosomia at birth and overweight/obesity at the 12th month, 24th month, and 36th month (Table 2). Children born to mothers with higher glucose level (≥7.8 mmol/L) at 24–28 gestational weeks had greater mean values of body weight and length at birth as well as higher prevalence of macrosomia at birth and overweight/obesity at the 12th month and 36th month compared with those born to mothers with normal glucose level (<7.8 mmol/L) at 24–28 gestational weeks.

After adjustment for variables in Model 1 or Model 3, there were positive associations of either maternal prepregnancy BMI or glucose level at 24–28 gestational weeks with the risks of macrosomia at birth and offspring's overweight/obesity at the 12th month, 24th month, and 36th month except for the nonsignificant positive association of maternal glucose level at 24–28 gestational weeks with the risks of offspring's overweight/obesity at the 24th month (Table 3). When maternal prepregnancy BMI and glucose level at 24–28 gestational weeks were entered into the multivariable-adjusted models simultaneously, these positive associations of maternal prepregnancy BMI with the risks of offspring's overweight status remained to be significant, while the positive association of maternal level at 24–28 gestational weeks with the risks of offspring's overweight/obesity at the 12th month, 24th month, and 36th month tended to be attenuated (Models 2 and 4, Table 3).

When maternal prepregnancy BMI and glucose level at 24–28 gestational weeks were simultaneously entered into the models as continuous variables, the multivariable ORs (95% confidence intervals) of macrosomia at birth and offspring's overweight/obesity at the 12th month, 24th month, and 36th month were 1.12 (1.11–1.13), 1.05 (1.04–1.06), 1.07 (1.06–1.08), and 1.11 (1.10–1.12) for each 1-unit increase (kg/m^2^) in maternal prepregnancy BMI and 1.13 (1.10–1.17), 1.01 (0.99–1.03), 0.99 (0.96–1.01), and 1.00 (0.97–1.02) for each 1-unit increase (mmol/L) in maternal glucose level at 24–28 gestational weeks, respectively (Table 3).

When mothers were further stratified into prepregnancy normal weight (BMI < 24 kg/m^2^) and overweight (BMI ≥ 24 kg/m^2^) groups, the positive association of high maternal glucose level at 24–28 gestational weeks with macrosomia at birth was similar between the two groups; however, the positive association of high maternal glucose level at 24–28 gestational weeks with childhood overweight risk at 12th and 36th months was only seen among prepregnancy normal weight mothers but not among overweight mothers (Table 4).

**Figure 1:**
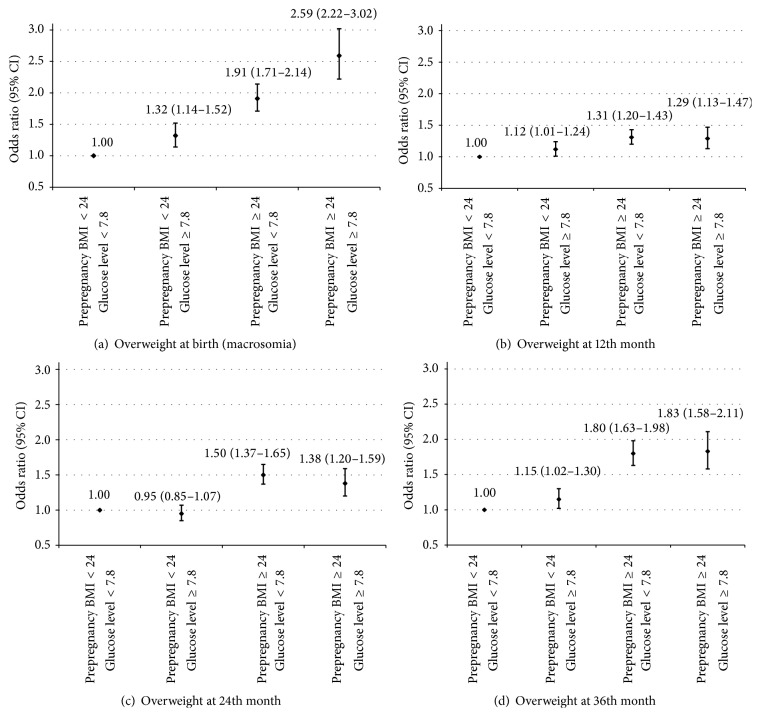
Odds ratios (95% CIs) of offspring's overweight status from birth to three years of age according to joint categories of maternal prepregnancy body mass index and glucose level at 24–28 weeks of gestation. Adjusted for maternal age, ethnicity, education, smoking status, history of abortion, systolic blood pressure at 24–28 gestational weeks, gestational age at delivery, and child sex in macrosomia analyses and additionally for birthweight and mode of infant feeding in childhood overweight/obesity analyses.

We additionally examined the joint association of maternal prepregnancy BMI and glucose level at 24–28 gestational weeks with offspring's overweight status (Figure 1). We used two categories of maternal prepregnancy BMI (<24 and ≥24 kg/m^2^) and two categories of maternal glucose level at 24–28 gestational weeks (<7.8 and ≥7.8 mmol/L). The positive associations between prepregnancy BMI and the risks of macrosomia at birth and offspring's overweight status at the 12th, 24th, and 36th month were found among mothers with glucose level < 7.8 mmol/L and ≥7.8 mmol/L. Similarly, the positive associations between maternal glucose level and the risks of macrosomia at birth and offspring's overweight status at the 36th month were found among mothers with prepregnancy BMI < 24 kg/m^2^ and BMI ≥ 24 kg/m^2^. Compared with children born to mothers with prepregnancy BMI < 24 kg/m^2^ and glucose level < 7.8 mmol/L, children born to mothers with prepregnancy BMI ≥ 24 kg/m^2^ and glucose level ≥ 7.8 mmol/L were at the highest risk to be macrosomic at birth and overweight/obesity at the 36th month of age.

## 4. Discussion

The present study indicated that offspring born to mothers with prepregnancy overweight/obesity or high glucose level at 24–28 gestational weeks were independently associated with increased risks of macrosomia at birth compared with those born to mothers with prepregnancy normal weight and normal glucose level. Our results suggested that maternal prepregnancy BMI plays a more important role than glucose level at 24–28 weeks of gestation with regard to childhood overweight at 1–3 years of age.

Previous studies have compared the relative impact of maternal prepregnancy obesity and hyperglycemia during pregnancy on the risk of macrosomia. Ehrenberg et al. found that maternal prepregnancy BMI > 30 kg/m^2^ would increase the risk of large-for-gestational-age (LGA) newborns by 60% compared with maternal prepregnancy BMI 19.8–25 kg/m^2^, while the risk of LGA newborns of prediabetic mothers was 4.4 times higher compared with those of mothers without diabetes [6]. They argued that since maternal prepregnancy overweight and obesity were more common than maternal diabetes during pregnancy, abnormal maternal body shape played a stronger role in LGA compared to maternal GDM during pregnancy. However, they did not directly compare the relative impact of maternal prepregnancy BMI and glucose level during pregnancy on their offspring's obesity risk at early life. In 2014, Liu et al. suggested that high BMI measured at GDM screening was the most important determinant for the risk of macrosomia [17]. However, this study only focused on macrosomia with no data on childhood overweight status. The present study concluded for the first time that maternal prepregnancy overweight played a more important role than glucose level during pregnancy with regard to offspring's overweight status at 1–3 years of age.

Our team previously found that both higher maternal fasting glucose at the first trimester and higher maternal OGTT glucose levels at the second trimester were associated with greater birth weight and birth length, less weight gain and length gain in the first 3 months of life, and more weight gain in months 6–12 of life [11, 12]. Similarly, the present study found that maternal glucose level during pregnancy was significantly associated with macrosomia at birth, but the positive association of maternal glucose level during pregnancy with offspring's overweight risk attenuated in the next three years. The results suggested that the effects of maternal high glucose levels during pregnancy on offspring's growth might differ at different ages. For prepregnancy BMI, besides the significant association with macrosomia at birth, prepregnancy overweight/obesity also significantly increased the risk of offspring's overweight after birth and this association became stronger with the growth of offspring. As obesity is the result of environmental and genetic interactions, we proposed a hypothesis that there might be a disparity of maternal prepregnancy BMI and glucose level during pregnancy on offspring's overweight status between birth and childhood. Fetal growth depends on intrauterine environment which is affected by both prepregnancy BMI and glucose level during pregnancy, while childhood growth depends on children's own lifestyle as well as genetic factors reflected by maternal prepregnancy BMI. This hypothesis needs to be confirmed by further studies.

As previous studies have demonstrated that both maternal prepregnancy overweight/obesity and GDM could increase the offspring's overweight risk [6–10], it is still important to confirm which strategy is more critical to prevent neonatal and childhood obesity, prepregnancy weight control or GDM prevention. The present study concluded that the positive association of maternal glucose level during pregnancy with childhood overweight risk was only seen among prepregnancy normal weight mothers but not among overweight mothers. Thus, our study suggested that the intervention strategy should be started from maternal prepregnancy weight control among overweight/obese mothers and glucose control during pregnancy among normal weight mothers.

The major strengths of our study include the perinatal cohort with more than 20 thousand mother-child pairs, repeated direct measures of the growth and development of offspring from birth to 3 years of age, and a wide range of potential confounders. Our study also has some limitations. First, about half of the women screened were excluded as they lack some of the key information. Though we compared the general characteristic between the included and excluded women and found that women excluded were older, we cannot exclude the possibility that the observed effect sizes in our study had departure from the true value. Second, we only had one time point of maternal glucose measurement during pregnancy, and thus future studies are needed to evaluate the association of glucose level from the oral glucose tolerance test (OGTT), which is the standard diagnostic method for GDM, with offspring's overweight status.

## 5. Conclusion

Our study indicated that maternal prepregnancy overweight/obesity and high glucose level at 24–28 gestational weeks were independently associated with the risks of macrosomia at birth and overweight among children in the first three years of life. The impact of maternal gestational hyperglycemia on offspring's overweight before 3 years of age can be modified by prepregnancy BMI.

## Supplementary Material

There was significant association between childhood obesity/overweight and mode of feeding. Children who fed on exclusive breast milk were less likely to be overweight, while children fed on exclusive formula milk were more likely to be overweight.

## Figures and Tables

**Table 1:** Characteristics of 21,354 mother-infant pairs according to different categories of maternal prepregnancy body mass index and glucose level at 24–28 gestational weeks.

	Prepregnancy body mass index (kg/m^2^)				*p* value	Glucose level (mmol/L)		*p* value
	<18.5	18.5–23.9	24.0–27.9	≥28		<7.8	≥7.8	
Number of subjects	2,394	13,602	4,055	1,303	—	17,321	4,033	—
*Maternal characteristics*								
Maternal age, y	27.1 (2.7)	27.9 (2.9)	28.2 (3.0)	28.3 (3.2)	<0.001	27.8 (2.9)	28.5 (3.1)	<0.001
Ethnicity (Han), %	95.9	95.8	95.7	96.0	0.98	95.7	96.3	0.079
Mother's education, %					<0.001			0.97
≤12 years	25.8	23.1	28.6	39.5		25.5	25.5	
>12 years	74.2	76.9	71.4	60.5		74.5	74.6	
Smoking during pregnancy, %	2.13	2.02	2.15	2.76	0.36	2.17	1.81	0.15
History of abortion, %	30.3	30.7	34.4	36.8	<0.001	31.1	34.5	<0.001
Prepregnancy body mass index, kg/m^2^	17.6 (0.8)	21.1 (1.5)	25.6 (1.1)	30.5 (2.4)	<0.001	21.9 (3.3)	23.2 (3.7)	<0.001
Systolic blood pressure at 24–28 gestational weeks, mmHg	103 (10)	107 (10)	111 (10)	115 (11)	<0.001	107 (10)	109 (11)	<0.001
Glucose level at 24–28 gestational weeks, mmol/L	6.25 (1.3)	6.50 (1.5)	6.92 (1.6)	7.27 (1.8)	<0.001	6.05 (1.0)	8.95 (1.2)	<0.001
*Child characteristics*								
Gestational age at delivery, weeks	39.6 (1.4)	39.6 (1.4)	39.6 (1.6)	39.4 (1.7)	<0.001	39.6 (1.4)	39.4 (1.6)	<0.001
Boy, %	49.5	52.0	51.6	50.6	0.14	51.6	51.5	0.95
Mode of infant feeding, %					<0.001			0.43
Exclusive breast-feeding	16.0	16.2	14.9	12.2		15.7	15.5	
Mixed breast and formula	36.7	35.4	38.4	41.6		36.4	37.0	
Weaned from breast-feeding	45.3	46.2	43.7	42.9		45.6	44.8	
Exclusive formula feeding	2.1	2.1	3.0	3.3		2.3	2.7	

Data are means (SD) or percentage.

**Table 2:** Percentage of macrosomia at birth or childhood overweight and obesity during early life according to different categories of maternal prepregnancy body mass index and glucose level at 24–28 gestational weeks.

	Prepregnancy body mass index (kg/m^2^)				*p* value	Glucose level at 24–28 gestational weeks (mmol/L)		*p* value
	<18.5	18.5–23.99	24.0–27.99	≥28		<7.8	≥7.8	
Number of subjects	2,394	13,602	4,055	1,303	—	17,321	4,033	—
*Weight*								
Birth, g	3,252 (407)	3,394 (441)	3,478 (491)	3,551 (529)	<0.001	3,391 (450)	3,456 (491)	<0.001
12th month, kg	10.1 (1.1)	10.4 (1.1)	10.6 (1.2)	10.7 (1.2)	<0.001	10.4 (1.2)	10.4 (1.1)	0.282
24th month, kg	12.5 (1.3)	13.0 (1.4)	13.2 (1.5)	13.5 (1.6)	<0.001	13.0 (1.5)	13.0 (1.5)	0.021
36th month, kg	14.7 (1.7)	15.3 (1.9)	15.7 (2.1)	16.3 (2.4)	<0.001	15.3 (2.0)	15.4 (2.0)	0.003
*Length/height, cm*								
Birth	49.9 (1.5)	50.2 (1.7)	50.4 (1.9)	50.5 (1.8)	<0.001	50.2 (1.7)	50.3 (1.7)	<0.001
12th month	76.5 (2.6)	77.0 (2.7)	77.1 (2.7)	77.2 (2.7)	<0.001	77.0 (2.7)	76.9 (2.6)	0.003
24th month	88.4 (3.3)	88.9 (3.2)	89.0 (3.2)	89.3 (3.2)	<0.001	88.9 (3.2)	88.8 (3.1)	0.132
36th month	97.6 (3.7)	98.2 (3.7)	98.5 (3.8)	99.1 (3.7)	<0.001	98.2 (3.8)	98.2 (3.6)	0.41
*Overweight status, %*								
Birth					<0.001			<0.001
Macrosomia	4.34	9.11	14.3	20.6		9.57	13.3	
12th month					<0.001			0.002
Overweight	16.3	18.1	19.8	20.3		18.1	19.5	
Obesity	9.98	15.0	19.2	22.2		15.4	16.8	
24th month					<0.001			0.072
Overweight	10.8	14.6	17.1	20.8		15.1	15.0	
Obesity	5.35	9.10	13.6	18.2		9.83	11.0	
36th month					<0.001			<0.001
Overweight	6.93	10.6	14.4	15.6		11.1	11.8	
Obesity	3.84	7.40	11.8	19.7		8.20	10.3	

Data are means (SD) or percentage.

Overweight was defined as body mass index ≥ 85th percentile (≥1.035 *Z* score); obesity was defined as body mass index ≥ 95th percentile (≥1.645 *Z* score); sex specific body mass index percentiles were based on WHO growth reference (World Health Organization, 2006).

**Table 3:** Odds ratio and 95% confidence interval of macrosomia at birth or childhood overweight/obesity during early life according to different categories of maternal prepregnancy body mass index and glucose level at 24–28 gestational weeks.

	Prepregnancy body mass index (kg/m^2^)				BMI as a continuous variable (1-unit increase)	Glucose level (mmol/L)		Glucose level as a continuous variable (1-unit increase)
	<18.5	18.5–23.99	24.0–27.99	≥28		<7.8	≥7.8	
Number of subjects	2,394	13,602	4,055	1,303	21,354	17,321	4,033	21,354
Macrosomia at birth								
Model 1	0.47 (0.38–0.58)	1.00	1.64 (1.47–1.82)	2.51 (2.15–2.93)	1.13 (1.12–1.14)	1.00	1.43 (1.28–1.59)	1.18 (1.14–1.21)
Model 2	0.49 (0.39–0.60)	1.00	1.56 (1.40–1.74)	2.31 (1.97–2.70)	1.12 (1.11–1.13)	1.00	1.27 (1.14–1.42)	1.13 (1.10–1.17)
Overweight/obesity at 12th month								
Model 3	0.78 (0.70–0.87)	1.00	1.21 (1.11–1.32)	1.33 (1.17–1.53)	1.05 (1.04–1.06)	1.00	1.10 (1.01–1.19)	1.02 (1.00–1.04)
Model 4	0.78 (0.70–0.88)	1.00	1.21 (1.11–1.32)	1.33 (1.16–1.52)	1.05 (1.04–1.06)	1.00	1.06 (0.97–1.15)	1.01 (0.99–1.03)
Overweight/obesity at 24th month								
Model 3	0.69 (0.61–0.79)	1.00	1.30 (1.18–1.42)	1.90 (1.66–2.18)	1.07 (1.06–1.08)	1.00	0.98 (0.90–1.07)	1.01 (0.98–1.03)
Model 4	0.69 (0.6–0.79)	1.00	1.30 (1.19–1.43)	1.91 (1.67–2.20)	1.07 (1.06–1.08)	1.00	0.92 (0.84–1.01)	0.99 (0.96–1.01)
Overweight/obesity at 36th month								
Model 3	0.60 (0.51–0.70)	1.00	1.51 (1.37–1.66)	2.25 (1.95–2.59)	1.11 (1.09–1.12)	1.00	1.16 (1.05–1.28)	1.03 (1.00–1.06)
Model 4	0.60 (0.51–0.70)	1.00	1.50 (1.36–1.66)	2.24 (1.94–2.59)	1.11 (1.10–1.12)	1.00	1.06 (0.96–1.17)	1.00 (0.97–1.02)

Model 1 adjusted for maternal age, ethnicity, education, smoking status, history of abortion, systolic blood pressure at 24–28 gestational weeks, gestational age at delivery, and child sex.

Model 2 adjusted for variables in Model 1 and also for glucose level (in prepregnancy BMI analyses) or prepregnancy BMI (in glucose level analyses).

Model 3 adjusted for variables in Model 1 and also for birthweight and mode of infant feeding.

Model 4 adjusted for variables in Model 2 and also for birthweight and mode of infant feeding.

**Table 4:** Odds ratio and 95% confidence interval of macrosomia at birth or overweight/obesity during early life according to different categories of glucose level stratified by prepregnancy BMI.

	Glucose level (mmol/L)		Glucose level as a continuous variable (1-unit increase)
	<7.8	≥7.8	
*Prepregnancy BMI < 24 kg/m* ^*2*^			
Number of subjects	13,395	2,601	15,996
Macrosomia at birth	1.00	1.31 (1.14–1.51)	1.15 (1.11–1.19)
Overweight/obesity at 12th month	1.00	1.12 (1.01–1.24)	1.02 (1.00–1.05)
Overweight/obesity at 24th month	1.00	0.95 (0.85–1.07)	1.00 (0.97–1.03)
Overweight/obesity at 36th month	1.00	1.14 (1.01–1.29)	1.02 (0.99–1.06)
*Prepregnancy BMI ≥ 24 kg/m* ^*2*^			
Number of subjects	3,926	1,432	5,358
Macrosomia at birth	1.00	1.36 (1.16–1.61)	1.16 (1.11–1.21)
Overweight/obesity at 12th month	1.00	0.99 (0.86–1.14)	0.99 (0.96–1.03)
Overweight/obesity at 24th month	1.00	0.92 (0.79–1.07)	0.99 (0.95–1.03)
Overweight/obesity at 36th month	1.00	1.03 (0.88–1.20)	0.99 (0.95–1.03)

Adjusted for maternal age, ethnicity, education, smoking status, history of abortion, systolic blood pressure at 24–28 gestational weeks, gestational age at delivery, and child sex in macrosomia analyses and additionally for birthweight and mode of infant feeding in childhood overweight/obesity analyses.

## Additional Points

*What Is Known on This Subject?* Maternal prepregnancy overweight/obesity increases the risks of poor fetal outcomes including macrosomia at birth and overweight at childhood. GDM is another independent risk factor for offspring's macrosomia at birth.* What Does This Study Add?* This study compared directly the relative impact of maternal obesity and hyperglycemia on the risk of macrosomia at birth and childhood overweight status at 0–3 years of age. It suggested that maternal prepregnancy overweight might play a more important role than glucose level during pregnancy with regard to offspring's overweight status at 1–3 years of age. The impact of maternal gestational hyperglycemia on offspring's overweight before 3 years of age can be modified by prepregnancy BMI.
