# Supplementary material for: Maternal Prepregnancy BMI and Glucose Level at 24–28 Gestational Weeks on Offspring's Overweight Status within 3 Years of Age
Source: Biomed Res Int. 2017 Jan 30;2017:7607210. doi: 10.1155/2017/7607210 (PMC5303844; doi:10.1155/2017/7607210)
Supplement: Supplementary file 1 — There was significant association between childhood obesity/overweight and mode of feeding. Children who fed on exclusive breast milk were less likely to be overweight, while children fed on exclusive formula milk were more likely to be overweight. [file 7607210.f1.docx]

Supplementary table 1. Percentage of childhood overweight and obesity during early life according to different categories of infant feeding mode

| Childhood obesity/overweight | Mode of infant feeding, % | | | | | p |
| --- | --- | --- | --- | --- | --- | --- |
|  | Exclusive breast-feeding | | Mixed breast and formula | Weaned from breast-feeding | Exclusive formula feeding |  |
| 12^th^ month | 33.4 | 34.8 | | 33.1 | 36.7 | 0.114 |
| 24^th^ month | 22.2 | 26.1 | | 24.8 | 30.1 | <0.001 |
| 36^th^ month | 16.8 | 20.1 | | 20.0 | 24.0 | <0.001 |
